# New Carbamoyl Surface-Modified ZrO_2_ Nanohybrids for Selective Au Extraction from E-Waste

**DOI:** 10.3390/molecules28052219

**Published:** 2023-02-27

**Authors:** Sarah Asaad, Marwa Hamandi, Guilhem Arrachart, Stéphane Pellet-Rostaing, Serge Kimbel, Stéphane Daniele

**Affiliations:** 1 CP2M, ESCPE Lyon—Ctentre National de la Recherche Scientifique, Université Claude Bernard Lyon 1, UMR 5128, 69616 Villeurbanne, France; 2 ICSM, Université de Montpellier, CEA, CNRS, ENSCM, 30207 Marcoule, France; 3 WEEECycling, 76400 Tourville-les-Ifs, France

**Keywords:** ZrO_2_, nano-hybrids, adsorption, carbamoyl phosphonic acids, surface grafting, e-waste

## Abstract

Efficient and selective extractions of precious and critical metal ions such as Au(III) and Pd(II) were investigated using zirconia nanoparticles surface modified with different organic mono- and di-carbamoyl phosphonic acid ligands. The modification is made on the surface of commercial ZrO_2_ that is dispersed in aqueous suspension and was achieved by optimizing the Bronsted acid–base reaction in ethanol/H_2_O solution (1:2), resulting in inorganic–organic systems of ZrO_2_-L**^n^** (L**^n^**: organic carbamoyl phosphonic acid ligand). The presence, binding, amount, and stability of the organic ligand on the surface of zirconia nanoparticles were confirmed by different characterizations such as TGA, BET, ATR-FTIR, and ^31^P-NMR. Characterizations showed that all the prepared modified zirconia had a similar specific surface area (50 m^2^.g^−1^) and the same amount of ligand on the zirconia surface in a 1:50 molar ratio. ATR-FTIR and ^31^P-NMR data were used to elucidate the most favorable binding mode. Batch adsorption results showed that (i) ZrO_2_ surface modified with di-carbamoyl phosphonic acid ligands had the highest adsorption efficiency to extract metals than mono-carbamoyl ligands, and (ii) higher hydrophobicity of the ligand led to better adsorption efficiency. The surface-modified ZrO_2_ with di-N,N-butyl carbamoyl pentyl phosphonic acid ligand (ZrO_2_-L**^6^**) showed promising stability, efficiency, and reusability in industrial applications for selective gold recovery. In terms of thermodynamic and kinetic adsorption data, ZrO_2_-L**^6^** fits the Langmuir adsorption model and pseudo-second-order kinetic model for the adsorption of Au(III) with maximum experimental adsorption capacity q_max_ = 6.4 mg.g^−1^.

## 1. Introduction

The material compositions of e-waste, such as mobile phones, personal computers, printers, and televisions, are generally heterogeneous, complex, and dependent on the type of the product. It is estimated that their composition includes over 30 substances, including a mixture of ferrous metals, glass, plastics, base metals (copper, aluminum, etc.), toxic heavy metals (mercury, lead, chromium, etc.), precious metals (gold, silver, platinum group metals (PGM), and others. Due to issues related to toxicity, negative environmental impact as well as primary extraction, market price fluctuations, material scarcity, availability, and access to resources, e-waste recycling is considered essential to recover metals. This approach provides an important secondary source of metallic raw materials [1] and a way for some countries to secure their supply safely and sustainably. The recovery of precious metals such as Au and Pd from different matrices is a challenging task for a chemist. Due to their wide industrial applications and the increasing use of these elements in various industries under different complex formulations, it is always necessary to develop new methods and techniques for their respective extraction. The various commercially available methods include pyrometallurgy and hydrometallurgy. The latter has advantages such as flexibility of operations, clean working conditions, low equipment requirements, and low toxicity of by-products. The initial and crucial step in hydrometallurgy is to extract valuable metal species from solid waste using leaching reagents, such as concentrated hydrochloric acid, which is considered one of the most widely used leaching agents because it is inexpensive and less toxic. It yields the metals as chlorinated complexes in the acidic aqueous solution. Various hydrometallurgical operations have been tested for the recovery of these desired precious metals, such as cloud point extraction [2], liquid-liquid extraction [3], solvent extraction [4], precipitation [5], and solid phase extraction [6].

Solid phase extraction offers several advantages over other techniques and is widely applied in environmental applications for the separation of metal ions. The method is performed by allowing the target metal ion in the solution to bind to the surface of a solid material called an adsorbent through either an ion exchange mechanism or a chelation mechanism. Hybrid nanomaterials with organic–inorganic or bioinorganic architecture represent a promising field of fundamental research due to their remarkable new properties and their multifunctional character. Many types of such adsorbents have already been used and studied to extract Au, and/or Pd under different conditions, such as activated carbon pellets [7], silica-based adsorbents [8], graphene oxide [9], biosorbents [10], resins [11], polymeric materials [12], nanomaterials [13], modified metal oxides [14].

However, the separation of precious metal ions (such as Au, and Pd) remains problematic, due to their complex chemistry and overlapping properties, which presents a challenge. Solid–liquid adsorption appears also to be a promising technology to extract precious metals from leach solutions containing extremely low metal concentrations. Therefore, it is necessary to develop adsorbents that have a high affinity to improve metal selectivity and high specific surface area, contributing to high adsorption capacities and faster adsorption rates. At the same time, their stability and reusability is a crucial factor for industrial applications [15]. For this purpose, ZrO_2_ is an excellent support because it can be used under very harsh reaction conditions (in terms of pH or redox) without being modified and phosphonic acids exhibited very high surface affinities. Various zirconium dioxide-based nanomaterials have recently been used for the removal and photodegradation of toxic pollutants and metals, and they exhibit excellent responses due to their superior mechanical properties in many different biological and catalytic applications [16,17,18].

In our group, commercial ZrO_2_ nanoparticles surface-modified with thioctic acid (TOA) were studied as effective nano-adsorbents for extracting Pd and Au from waste electrical and electronic equipment (WEEE) water recycling processes [19]. The prepared ZrO_2_-TOA showed a remarkable selectivity for the recovery of precious metals (Pd and Au) quantitatively and at trace level (<10 ppm) from the complex mixture of Ni, Fe, Cu metals present in real e-wastewater. However, in strong acidic conditions (HCl > 1 M), the adsorption capacity was reduced due to the cleavage of the bonds between the carboxylic group of TOA and ZrO_2′_s surface. The problem was overcome by intercalating a phosphonic acid moiety (alendronic acid) between the ZrO_2_ surface and the thiotic acid via a two-step functionalization. A very recent publication on the functionalization of nano-TiO_2_ by mercatopropoyl phosphonic acid also demonstrates the advantage of using this type of ligand to improve the stability of the organic–inorganic system over a wide range of low pH [20]. The second issue we faced was the possible oxidation of the sulfur functions in a strongly oxidizing environment (presence of NO_3_^−^ ions) and thus the loss of affinity with the PGM metals. These motivated our search for ligands based on non-sulfur phosphonic acids for the surface modification of ZrO_2_ particles and for Au and Pd extractions.

We have recently developed bifunctional extracting molecules carrying an amide group and a phosphonic acid and/or a phosphonate group for the selective extraction of uranium(VI) versus iron(III) from phosphoric acid solutions [21,22]. These studies showed that the molecular engineering of these ligands, such as the nature of alkyl chains on the amide part, the spacer length between the two functionalities, and the degree of steric hindrance on the spacer of such carbamoyl phosphonic acid or carbamoyl phosphonate ligands, affected their efficiency for the extraction of uranium. It was found that a specifically designed ligand, butyl-1-[N,N-bis(2-ethylhexyl)carbamoyl]nonyl phosphonic acid (DEHCNPB), had high-efficiency structure because: (i) the amide group was alkylated by branched aliphatic chains (e.g., 2-ethylhexyl chain), (ii) amide and phosphoryl groups was separated by a methylene bridge, (iii) the methylene bridge has to be alkylated (typically by phenyl or an alkyl group), and (iv) a mono-alkyl phosphonic acid group was preferred to a phosphonic acid in order to improve the solubility and the behavior of the molecule during the solvent extraction process. DEHCNPB showed very selective extraction of uranium(VI) from phosphoric acid even in the presence of high concentrations of iron(III) and other elements in genuine industrial phosphoric acid solution with unequaled performance [23].

Given the highly promising extraction behavior with carbamoyl-phosphonic acid ligands and the stability properties of ZrO_2_ nanoparticles, we reported herein the study of the extraction of Au and Pd as precious metal ions using different families of carbamoyl phosphonic acid functionalized ZrO_2_ in chloride acid medium and at room temperature. The main objective of the project was to elucidate how the nature of the carbamoyl-phosphonic acid ligand could have an impact on the selectivity and the ability of the hybrid adsorbent to take up Au and/or Pd.

## 2. Results and Discussion

### 2.1. Selection of Trapping Ligands and Synthesis of Hybrid ZrO_2_ Nano-Adsorbent

Six ligands were selected from the phosphonic acid family with either one carbamoyl function (CO-NRR’) (Group A: L**^1^**–L**^4^**) or two carbamoyl functions (Group B: L**^5^** and L**^6^**) (Table 1). The specifications of the selected ligands included a robust anchoring function on the ZrO_2_ surface and a metal-specific trapping function. For the anchoring function, it was decided to favor phosphonic acids (R-PO_3_H_2_) which create strong interactions with ZrO_2_ and are used in very aggressive environments such as those containing concentrated acids, strong oxidants, or even radiolysis agents [24]. The mono-carbamoyl ligands studied will differ in the aliphatic chain length (and thus the hydrophobicity) of the amide function to verify if it will affect the performance. In the case of the di-carbamoyl ligands, it is the length (and thus the flexibility) of the chain between the phosphonic acid function and the di-carbamoyl group that will be tested.

The immobilization of the organic ligands on the surface of the zirconia nanoparticles (ZrO_2_-L**^n^**) was carried out by an acid–base reaction according to Bronsted between the phosphonic acid functions and the hydroxyl functions on the surface of ZrO_2_, aiming at a final ZrO_2_-L**^n^** ratio of 50. This ratio has already been investigated in our previous studies and has proven to be very promising [19] and will also allow us to benchmark the performances of the new nano-absorbents.

### 2.2. Characterizations

**Figure 1:**
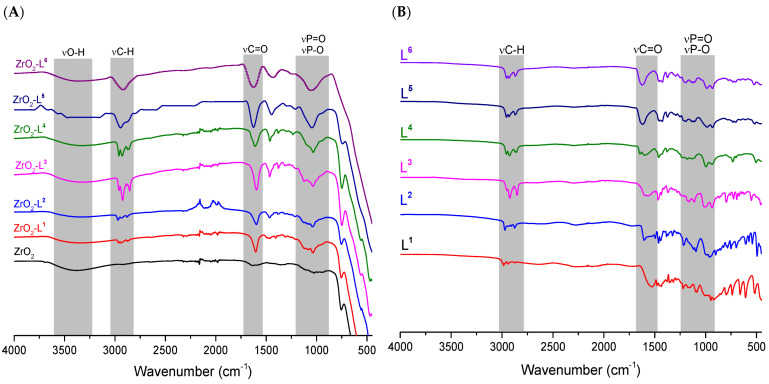
ATR-FTIR spectra of (**A**) ligands L**^n^** and (**B**) ZrO_2_-L**^n^** nanomaterials.

ATR-FTIR spectroscopy was used to identify the presence of ligands and determine relevant information on the nature of phosphonic acid bonds on the zirconia surface. All the spectra obtained from 400 to 4000 cm^−1^ (Figure 1) showed the same characteristics. Indeed, compared to pure ZrO_2_, the broad ν Zr-OH band centered around 3300 cm^−1^ decreased in intensity, confirming the interaction of the hydroxyl groups of zirconia with the phosphonate functions (PO_3_) of the ligands. The spectra also showed the appearance of the characteristic bands of the ligands, in particular the ν C-H around 2900 cm^−1^ and the ν P-O and ν P = O between 900 and 1400 cm^−1^ [25,26].

**Figure 2:**
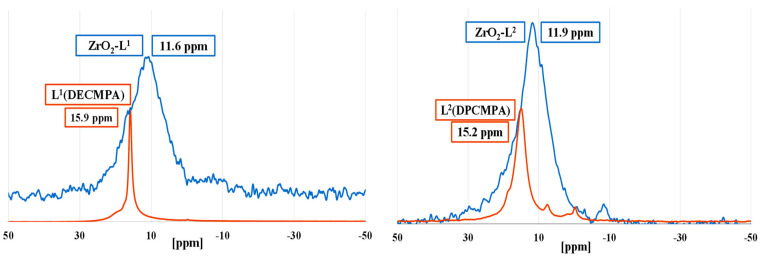

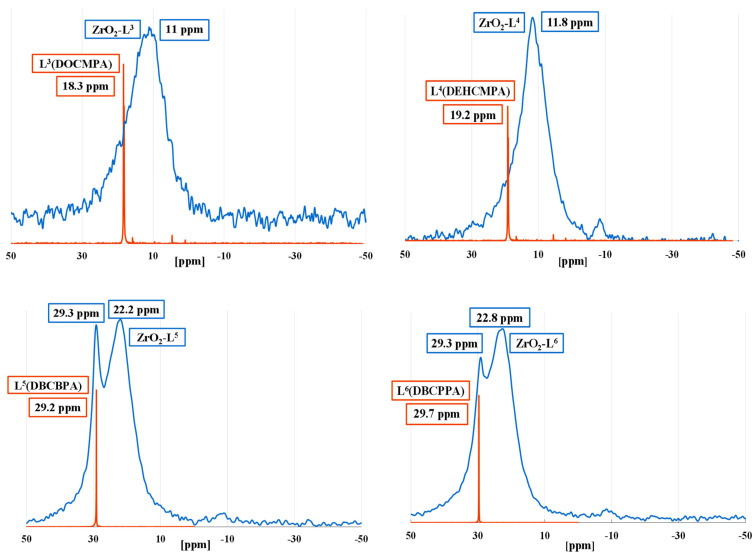
Solid-state ^31^P NMR spectra of ZrO_2_-L**^n^** nanomaterials (blue) and liquid-state ^31^P NMR spectra of ligands (red).

Solid state ^31^P NMR of modified nano ZrO_2_ with phosphonic acids organic ligands (L**^n^**) allowed to study the binding mode (monodentate, bidentate, or tridentate) of the phosphonic groups, and the different types of physisorbed and/or chemisorbed phosphonate species on the zirconia surface (Figure 2). While all liquid-state ^31^P NMR spectra of the organic phosphonic ligands showed very sharp peaks, ^31^P solid-state NMR spectra of all the hybrid zirconia exhibited broad resonance signals suggesting thus effective chemical anchoring to the ZrO_2_ surface. All ligands except di-carbamoyls displayed a single peak suggesting one major mode of grafting.

The shielding of the chemical shifts (5–7 ppm) between ZrO_2_-L**^n^** and free ligands demonstrated that the electron density is higher around the P nuclei of the supported ligand, which might be due to the charge of the phosphonate anion. The values of the chemical shift suggested that for mono-carbamoyl ligands, we had mostly a bidentate coordination mode. In the case of di-carbamoyl ligands, the presence of a peak with a chemical shift close to that of the ligand could indicate either a physisorption of certain ligands or a monodentate mode. Despite repeated washings, this peak is still present for ZrO_2_:L**^n^** (*n* = 5 or 6) ratios of 50. The absence of peaks with negative chemical shifts demonstrates the absence of molecular phosphonate complexes resulting from corrosion.

**Figure 3:**
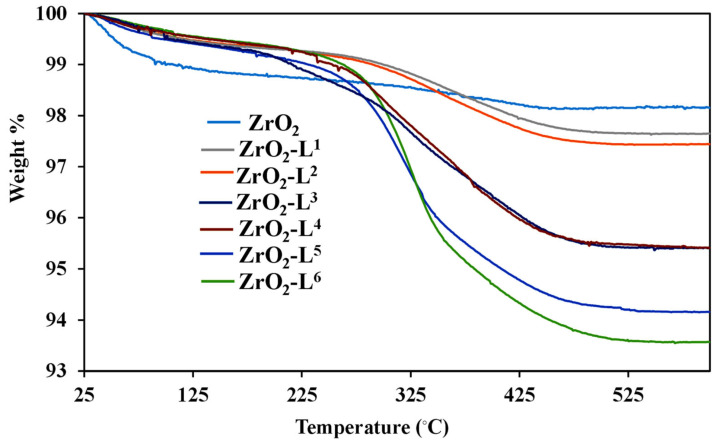
TGA curves of ZrO_2_ and ZrO_2_-L**^n^** samples.

The weight losses given by the TG analysis between 20 and 900 °C and under air (Figure 3) are summarized in Table 2 and were used to estimate the contents of ligands on the surface of hybrid ZrO_2_ powders and grafting densities. As shown in Figure 3, the first weight loss below 200 °C was attributed to the loss of residual solvents. All surface-modified ZrO_2_ exhibited a significant weight loss between 200 and 500 °C, representing the removal of remaining –OH and L**^n^** ligands on the zirconia surface. It should be noted that the phosphonate parts (PO_3_) were not totally eliminated and were still present on the surface after calcination at 900 °C in the form of phosphate. Nevertheless, the results showed that the theoretical and experimental weight losses between 200 and 500 °C are in agreement [19].

The grafting density is the number of grafted ligands per unit surface area.

For ZrO_2_-L**^n^** hybrid material, they were obtained from TGA data and using the following equation eq:

(1) $$GD\left(\frac{molecule}{nm^{2}}\right)=\frac{F_{L}{\;\times \;N}_{A}\;}{S\;\times \;M\;}\times 10^{21}$$

where F*_L_* is the fraction of the ligand on the surface of zirconia (% wt.), N*_A_* is Avogadro number, S is the specific area of zirconia (m^2^/g), and M is the molecular weight of the ligand (g/mol).

**Figure 4:**
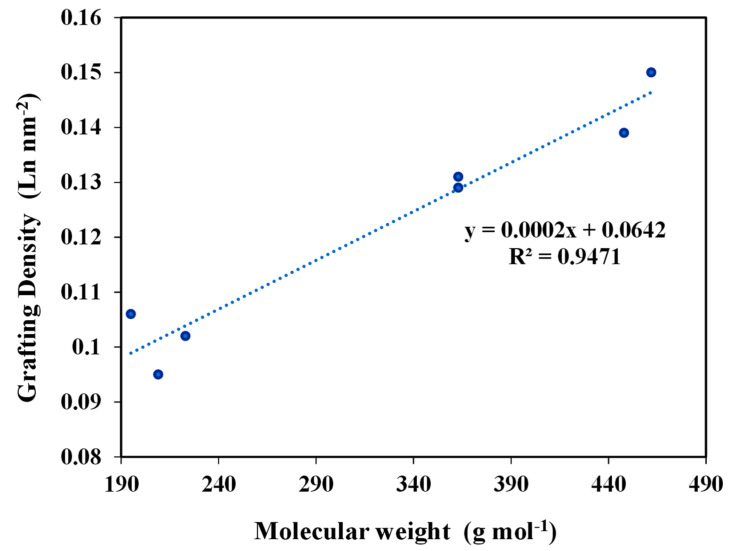
Variation of Grafting Density as a function of Molecular Weight of ligands.

The estimated grafting densities gave a range of 0.1–0.150 L**^1−6^**/nm^2^. In the case of the particles anchored under the standard conditions, these values suggested a coverage not exceeding the monolayer for the different prepared materials. Indeed, a linear proportional relation was observed between the grafting density of ligands and the molecular weight of these ligands (Figure 4). This also proved that the grafting of the ligands was not affected by the steric hindrance of the higher molecular weight ligands.

In order to quantify the amount of ligand on the surface, elemental phosphorus analyses showed that the amounts of phosphorus on the ZrO_2_ surface were identical for all nanomaterials (Table 2). Therefore, it can be estimated that the performance of the different hybrid nanomaterials in terms of sorption will not depend on the ligand content.

The BET analysis for all the prepared ZrO_2_-L**^n^** materials had a similar specific surface area (~50 m^2^/g). Even after functionalization, the surface area was preserved. The XRD spectrum (Figure S1) of commercial nonmodified zirconia (10% wt., 100 nm) showed two phases present in the powder, tetragonal (52%) and monoclinic (48%) with an estimated particle size of 12.7 nm and crystallinity of 84%. The diffraction pattern for modified ZrO_2_-L**^n^** shows similar indexes to non-modified ZrO_2_ for tetragonal and monoclinic with an estimated particle size of 12.1 nm. No additional peaks were observed after grafting the ligands onto the ZrO_2_ surface. TEM images (Figure S2) showed no morphology changes between non-modified ZrO_2_ and modified ZrO_2_-L**^n^** nanoparticles.

These data indicated that our grafting process did not modify the morphology and structure of the ZrO_2_ starting support as was also confirmed in our previous study [19], beneficial for the adsorption application.

### 2.3. Au and Pd Adsorption Studies

The adsorption studies were all carried out in the most acidic environment possible (pH < 3) in order to come as close as possible to the physicochemical conditions of industrial solutions. For this reason, no study was carried out concerning the pH.

**Figure 5:**
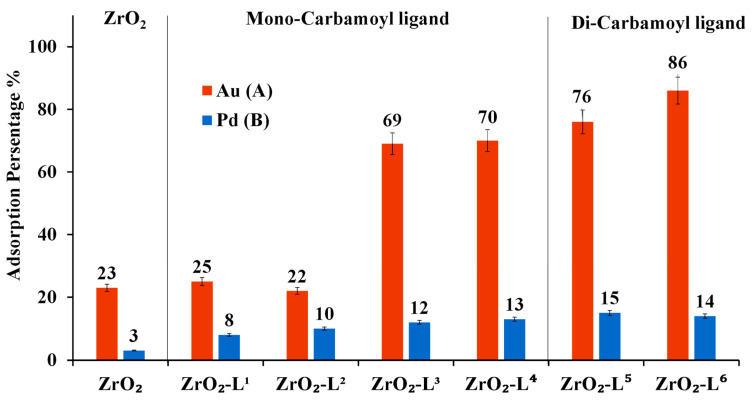
Adsorption percentage (%) of ZrO_2_-L**^n^** nanoparticles based on the single compound solution. (A) [Au(III)] or (B) [Pd(III)] = 10 ppm, V = 20 mL, m ZrO_2_/Ligand material = 50 mg, time = 24 h, Temp = 25^◦^C, pH ≈ 2.6.

As shown in Figure 5A, adsorption results of Au(III) showed that the di-carbamoyl ligands-modified nanoparticles promoted better Au(III) adsorption performance than mono-carbamoyl ligands-modified nanoparticles, and it was also noticed that the more hydrophobic mono-carbamoyl ligands-modified nanoparticles have a better adsorption activity than less hydrophobic ones. However, for the adsorption of Pd(II) as shown in Figure 5B, all carbamoyl ligand-modified ZrO_2_ nanoparticles show almost the same adsorption efficiency toward Pd(II).

The adsorption tests of Au(III) using modified zirconia nanoparticles have demonstrated that zirconia modified with Di(*N*,N butyl) carbamoyl pentyl phosphonic (ZrO_2_-L**^6^**) was found to be the best adsorbent for Au, which can be attractive to selectively separate these two elements. Our previous study based on thiotic acid ligand did not allow this selectivity [19].

Both the distribution coefficient (Kd) and separation factor values of Au compared to Pd metal ion using all prepared modified ZrO_2_-L**^n^** have been calculated (Table 3). The data confirm that Au metal ions are highly adsorbed by ZrO_2_-L**^6^** (exhibiting the highest Kd = 2.46 × 10^3^ mL g^−1^. Noticeably, ZrO_2_-L**^6^** displays a distinctive selectivity for the adsorption of Au compared to Pd (given the high SF of Au in comparison to Pd = 37.85).

**Figure 6:**
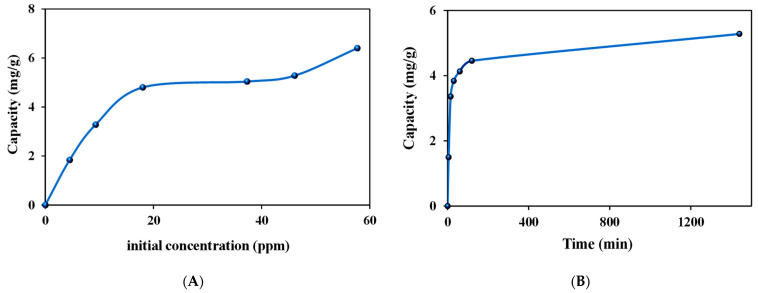
Au(III) capacities of ZrO_2_-L**^6^** as a function of (**A**) initial concentration and (**B**) contact time.

The possibility of separating gold from palladium via these nanomaterials encouraged us to study them in more detail for the extraction of gold from mixtures and especially with the L**^6^** ligand. The adsorption capacity (isotherm study), the effect of contact time (kinetic study) of ZrO_2_-L**^6^** toward Au(III), and the study of Au(III) stripping (desorption) and reusability of ZrO_2_-L**^6^** were investigated to understand adsorption characteristics and application capability of such hybrid ZrO_2_ nanoparticles (Figure 6).

From Figure 6A, the Au(III) data reflected a continuous increase in capacity as the initial Au(III) concentration increased in the range of 0–20 ppm. This indicated that all the active sites on the zirconia surface were sufficient for the adsorption process at the studied Au(III) initial concentrations. The maximum experimental adsorption capacity was determined to be 6.4 mg/g for Au(III) using ZrO_2_-L**^6^** nanoparticles.

The observed data were fitted to Langmuir and Freundlich isotherms, which described the relationship between the amounts of Au(III) adsorbed and its equilibrium concentration in solution. According to the isothermal Au adsorption data, the linear plots were constructed as shown in Figure S3a,b.

According to the Langmuir isotherm equation, a plot of 1/qe vs. 1/Ce was constructed, and according to the Freundlich isotherm equation, a plot of log qe vs. log Ce was also constructed. The values of the isotherm parameters were observed and compared as shown in Figure S3a,b and Table 4. From the observed data, the experimental data for Au(III) adsorption were found to fit the Langmuir model more since the correlation coefficient R^2^ was 0.9892 and the maximum calculated capacity of 6.54 mg/g was very close to the experimental capacity of 6.40 mg/g. There is thus strong evidence that the adsorption of Au(III) on ZrO_2_-L**^6^** occurred in a monolayer on a finite number of identical and specific adsorption sites with very low interaction between molecules in agreement with the Langmuir isotherm theory.

The Au(III) adsorption capacities on ZrO_2_-L**^6^** were examined from 5 to 1440 min to establish an optimum contact time between Au(III) ions and nano-adsorbents.

As shown in Figure 6B, it is obvious that the adsorption is very fast at the beginning, followed by a plateau phase. The highest Au(III) removal capacity by ZrO_2_-L**^6^** was achieved after 120 min of stirring. The experimental kinetic data for Au(III) adsorption on the prepared ZrO_2_-L**^6^** were fitted with pseudo-first-order and pseudo-second-order kinetic models to investigate the mechanism of each adsorption process. The kinetic parameters and correlation coefficients were calculated from the linear plots of log(qe-qt) versus t for the pseudo-first-order model, (t/qt) versus t for the pseudo-second-order model, as shown in the following Figure S4a,b and Table 5. Comparing the value of qe (experimentally) for the adsorption process, which was equal to 4.46 mg/g after 120 min, with the qe values (calculated) in the pseudo-second-order kinetic adsorption models, we concluded that the experimental adsorption values were closer to the qe values (calculated). This proves that the pseudo-second-order model correctly represented the adsorption mechanism, suggesting a chemisorption process.

### 2.4. ZrO_2_-L**^6^** Regeneration

The regeneration and reusability of modified nano-powder of ZrO_2_-L**^6^** were investigated for the Au(III) by adsorption/stripping cycles up to four times (20 mL of 10 ppm Au(III), 50 mg ZrO_2_-L**^6^**, 120 min adsorption contact time, and 4 h of stripping using a 10 mL mixture of 0.2 M thiourea and 0.5 M HCL, pH range 1–2). After each adsorption/stripping cycle, the ZrO_2_-L**^66^** powder was washed with 10 mL of water to be reused for other adsorption cycles.

**Figure 7:**
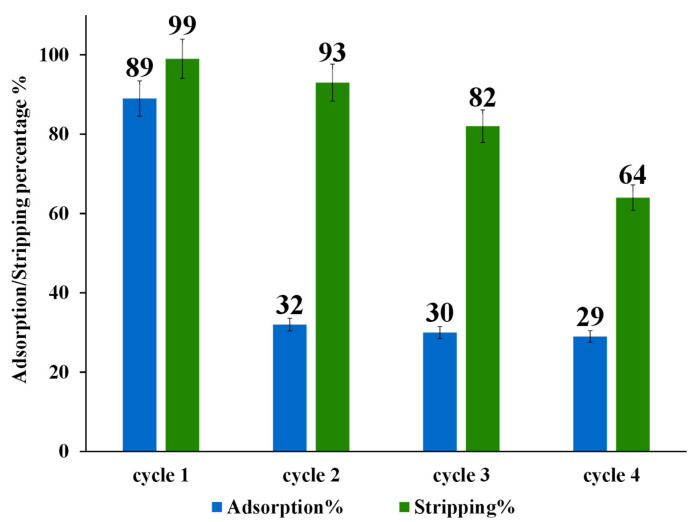
Adsorption and stripping cycles of 10 ppm Au(III) on ZrO_2_-L**^6^**.

As shown in Figure 7, the adsorption capability of ZrO_2_-L**^6^** decreased drastically after the first adsorption/stripping cycle from about 90% to a stable value of about 30%. The stripping percentage using 0.2 M thiourea and 0.5 M HCL obtained for four adsorption/desorption cycles displayed a moderate decrease after each cycle.

To understand the lack of reusability, modified ZrO_2_-L**^6^** and non-modified ZrO_2_ were characterized by CHNS elemental analysis before and after adsorption and stripping cycles. As shown in the following Table 6, commercial ZrO_2_ (entry 1) and as-prepared ZrO_2_-L**^6^** (entry 2) have some sulfur content (around 0.2 %wt) that could be due to the presence of sulfate ions on the surface of commercial ZrO_2_ samples. The ZrO_2_-L**^6^** sample before and after 5 adsorption and stripping cycles (Table 6, entry 3) showed the presence of a high amount of sulfur-based impurity (0.51 %wt) which can only come from thiourea contamination, being accompanied by a sharp drop in adsorption performance (Figure 7).

**Figure 8:**
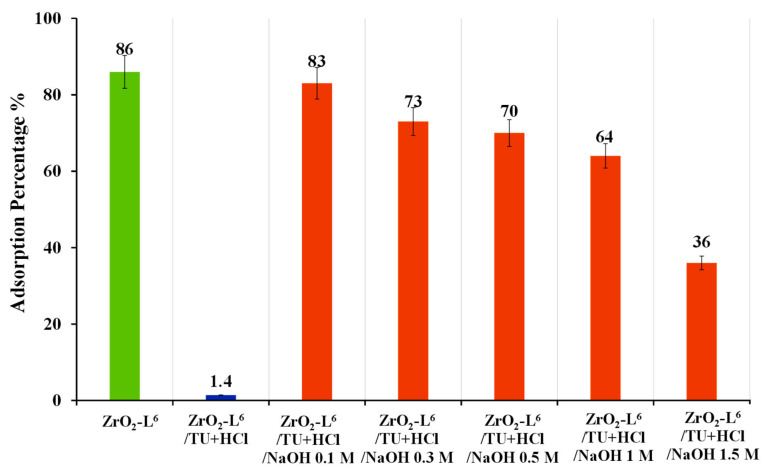
Optimization of washing step for the regeneration of ZrO_2_-L**^6^** after stripping based on adsorption percentage results of 10 ppm Au(III).

Adding a washing step to recover the adsorption activity for the modified ZrO_2_-L**^6^** was investigated. The commercial ZrO_2_ was washed with sodium hydroxide (NaOH 1 M) and analyzed with elemental analysis. The results (Table 6, entry 4) showed that washing with NaOH successfully led to the removal of these sulfur impurities. The modified ZrO_2_-L**^6^** was then treated with a stripping agent (ZrO_2_-L**^6^**/TU+ HCl) and washed with different concentrations of NaOH solution (0.1–1.5 M) and used for the adsorption of Au(III). As shown in Figure 8, the results showed that the adsorption efficiency of ZrO_2_-L**^6^** could be largely restored by integrating a wash phase with 0.1 M NaOH after the stripping phase.

**Figure 9:**
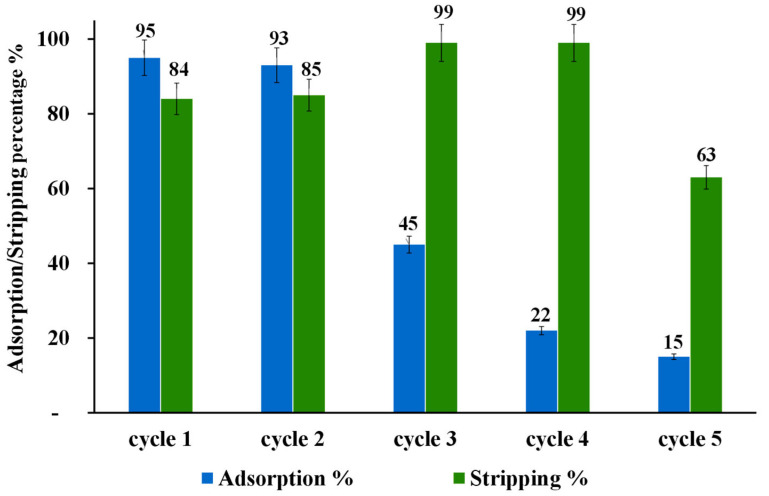
Effect of adding a 0.1 M NaOH washing step after each Au Adsorption/Stripping cycle using ZrO_2_-L**^6^**.

Figure 9 shows the results of regeneration and reusability of modified nano-powder of ZrO_2_-L**^6^** for the Au(III) by adsorption/stripping/washing cycles. They confirmed the regeneration of the adsorbent after washing with NaOH. However, the Au(III) adsorption efficiency is limited for two cycles due to the possible ligand degradation under basic conditions of washing with NaOH. CHNS elemental analysis of ZrO_2_-L**^6^** after 5 adsorption/stripping/washing cycles (Table 6, entry 5) confirmed the significant loss of nitrogen atoms and thus at least partial degradation of the ligand. Using sodium carbonate Na_2_CO_3_ 0.1 M as a weaker base for the washing step instead of NaOH gave the same results (Table 6, entry 6).

### 2.5. Selectivity of ZrO_2_-L**^6^** for Au(III) adsorption in real E-Waste Water

**Figure 10:**
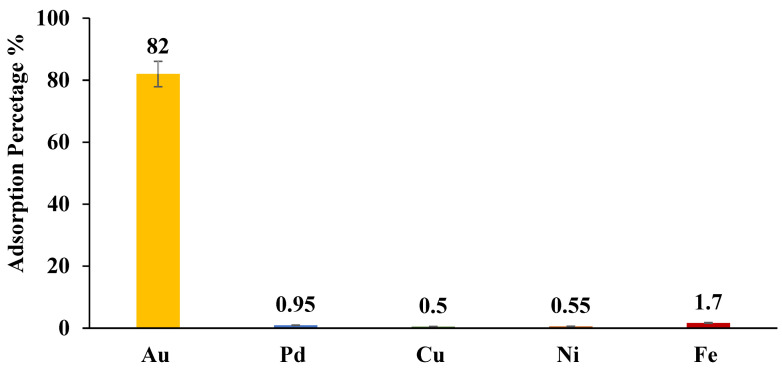
ZrO_2_-L**^6^** for adsorption of Au and other metals in real e-waste water solution.

The selectivity of ZrO_2_-L**^6^** towards 10 ppm Au(III) in real effluent (provided by WEEECycling) was investigated. Figure 10 shows that modified ZrO_2_-L**^6^** selectively extracted Au(III) (82 %) compared to other present metals Pd, Cu, Ni, and Fe, which were poorly removed (0.5 %–1.7 %) despite their high concentration in the initial real effluent (Pd = 6 mg/kg, Cu = 7918 mg/kg, Ni = 200 mg/kg, Fe = 540 mg/kg) (Table 7). Values reveal a strong affinity of the modified ZrO_2_-L**^6^** (DBCPPA) surface sites toward Au(III) (Kd = 1.82.10^3^ mL g^−1^), high selectivity (SF_Au/Pd, Cu, Ni, or Fe_ = 260–910), and very strong stability in the highly acidic medium of real e-wastewater.

## 3. Materials and Methods

### 3.1. Chemicals and Reagents

(*N*,*N*)-dioctylcarbamoylmethyl phosphonic acid (DOCMPA), (*N*,*N*)-bis(2-ethylhexyl) carbamoyl methyl phosphonic acid (DEHCMPA), (*N*,*N*)-diethyl carbamoyl methyl phosphonic acid (DECMPA), (*N*,*N*)- dipropylcarbamoylmethyl phosphonic acid (DPCMPA) and other ligands including Di(*N*,*N*-butylcarbamoyl)butyl phosphonic acid (DBCBPA), and Di(*N*,*N*-Butycarbamoyl)Pentyl phosphonic acid (DBCPPA) were synthesized according to the procedure described in the literature [21,22,27] and provided by Institut de Chimie Séparative de Marcoule (ICSM).

ZrO_2_ nanosuspension (10 %wt in H_2_O, pH 4–5, dp < 100 nm) and ICP Standard solutions of AuCl_3_ (999 ± 2 mg/kg in 5 % HCl TraceCERT), and PdCl_2_ (999 ± 2 mg/kg in 5% HCl TraceCERT) were purchased from Sigma-Aldrich (Saint6Quentin-Fallavier, France).

Ethanol (C_2_H_5_OH, ACS reagent, 100%), thiourea (NH_2_CSNH_2_), sodium hydroxide (NaOH,) and hydrochloric acid (HCl, ACS reagent, >37 %) were purchased from Sigma-Aldrich.

Real effluent of e-wastewater (HCL > 1 M, Au = 10 mg/kg, Pd = 6 mg/kg, Cu = 7918 mg/kg, Ni = 200 mg/kg, Fe = 540 mg/kg) was provided by WEEECycling company (Tourville-les-IfsFrance).

All solutions were prepared using deionized 18 MΩ.cm water and all chemicals were of analytical grade and used without further purification.

### 3.2. Equipment and Characterization

The estimation of organic content on the ZrO_2′_s surface was carried out using the TGA/DSC 1 STARe system from Mettler Toledo in crucibles alumina 70 µL. The experiments were recorded under air, and the crucibles were heated from 25 °C to 900 °C at a rate of 10 °C.min^−1^.

Textural properties were investigated by determining the specific surface areas of materials using the Micrometrics ASAP 2020 system, which is based on the BET method. Before the measurements, solids were desorbed at 423 K for 3 h. The XRD analysies were carried out for the identification of commercial and non-modified ZrO_2_ (10 % wt., 100 nm) and synthesized modified ZrO_2_-L**^n^**. Powder samples were analyzed in a horizontal position on D8 advanced from Brucker (Wissembourg, France), the diffraction was carried out according to Bragg’s law. Anticathode cooper X-ray sources were used with photons of a wavelength of 1.5460 Ǻ. Transmission electron microscope (TEM) JEOL 1400 F (120 kV) at Center Technologies of Microstructure (Villeurbanne, France), was used to study morphology of commercial non-modified ZrO_2_ and prepared modified ZrO_2_-L**^n^**.

Functionalization of surface-modified nano-zirconia materials was determined using attenuated total reflection ATR-FTIR on Thermo scientific system (model Nicolet Avatar 380) equipped with Diamond crystal (working range: 4000–400 cm^−1^). The liquid-state ^31^P NMR spectra of organic ligands were obtained by dissolving the water-soluble ligands (L**^1^**, L**^2^**) in deuterium oxide (D_2_O), and ethanol-soluble ligands (L**^3^**, L**^4^**, L**^5^**, L**^6^**) in methanol-D4. The solid-state ^31^P NMR spectra of hybrid zirconia modified were obtained on a Bruker AVANCE III 500 WB NMR system at a resonant frequency of 121.5 MHz using a 4 mm triple H/X/Y probe and a spinning speed of 10 kHz. All measurements were set to H_3_PO_4_ 85 % as a reference.

The contents of zirconium and phosphorus in the materials were determined by Elemental analyses ICP-OES at IRCELYON (Villeurbanne, France). Metal (Au, Cu, Ni, Fe, and Pd) elemental analyses were carried out by ICP-OES at Institut de Chimie Séparative de Marcoule (ICSM,). Direct determinations were carried out using suitable emission lines (Pd at 340.458, 324.270 nm, Au at 242.795, 267.595, 208.209 nm, Cu at 327.396, 224.700, 219.958, 217.894 nm, Ni at 221.647, 231.604, 341.476, 232.003 nm and Fe at 259.940, 238.204, 240.488, 371.994 nm).

### 3.3. Direct Surface Modification Process

The first step was to optimize the synthesis conditions and in particular the volume and the nature of the reaction solvent. The synthesis in water/alcohol media using a high amount of ethanol as a solvent for some ligands (L**^3^**–L**^6^**) (6 mL water/10 mL ethanol) led to low yields (20 %). To reduce this corrosion of ZrO_2_, ligands were dissolved in 1 mL of ethanol for the non-water-soluble ligands (L**^4^**–L**^6^**). The ligand solution was then added dropwise to the aforementioned ZrO_2_ suspended solution and the mixture was kept under stirring for 24 h at room temperature. ZrO_2_ we performed the syntheses in (6 mL water/1 mL ethanol) which led to yields of the order of 90 % concerning ZrO_2_.

The optimized surface functionalization step is: 6 mL (5 mmol of ZrO_2_) of a 10 wt% aqueous suspension at pH 4–5 was added to a 25 mL round bottom flask. A 0.1 mmol solution of each organic ligand (L**^n^**) was prepared by dissolving a different amount of each organic ligand in either 3 mL of water for ligand soluble in water at room temperature. The ZrO_2_ to ligand molar ratio was set at 50:1. Centrifugation (10,000 rpm for 10 min) was applied to separate the ZrO_2_/Ligand solid phase. In addition, the resulting solid was dispersed and washed three times with 10 mL water and three times with 10 mL ethanol. Centrifugation was applied after each washing step. Finally, the ZrO_2_-L**^n^** hybrid nanomaterials were dried at 70 °C for 24 h to obtain yields of about 80–97%.

### 3.4. Adsorption Experiments

Batch adsorption experiments were investigated by mixing 50 mg of different hybrid Nanopowders in a 50 mL glass bottle containing 20 mL of 10 ppm aqueous *M*Cl_3_ (*M* = Au, Pd) solution at pH range 2–3. The mixtures were stirred by a magnetic stirrer for 24 h at 25 °C (Room Temperature). The supernatant metal solutions were separated by centrifugation (10,000 rpm for 10 min), and the remaining metal concentrations were determined using ICP-OES. The adsorption or extraction percentages E (%) of Au, or Pd were calculated as follows:

(2) $$E\left(\%\right)=\frac{\left(C_{i}-C_{f}\right)}{C_{i}}\times 100\;\%$$

where C*_i_* (ppm) and C*_f_* (ppm) are the metal concentrations in the solution before and after the adsorption process, respectively.

To indicate the selectivity of the prepared sorbents towards Au relative to other competing metal ions (M) in solution, the distribution coefficient (K_d_, mL g^−1^) was calculated according to Equation (3):

(3) $$K_{d}=\frac{\left(C_{i}-C_{t}\right)}{C_{t}}\times \frac{V}{m}\;\;$$

where V is the volume of the solution (mL), m (g) is the amount of sorbent, and C*i* and C*t* are the initial metal concentration and the concentration at time t (min), respectively. The separation factor was also (SF_Au_, M) calculated, and is determined as follows Equation (4):

(4) $$\mathrm{SF}\left(\mathrm{Au},\;M\right)=\frac{K_{d}^{Au}\;}{K_{d}^{M}}\;\;\;$$

where K^ᴬᵘ^_d_ and Kᴹ_d_ are the distribution coefficients of Au and a competing ion M, respectively.

The adsorption capacity of ZrO_2_-L**^6^** was studied by mixing 20 mL of Au solution (increasing initial concentration in the range of 5 ppm to 60 ppm) with 25 mg of ZrO_2_-L**^6^**. The mixture was continuously stirred by a magnetic stirrer for 24 h at 25 °C. Capacity (q mg.g^−1^) was calculated as follows Equation (5):

(5) $$q\;\left(\frac{mg}{g}\right)=\frac{\left(C_{f}-C_{i}\right)\;\times \;V}{m}$$

where C*_i_* and C*_f_* are the metal concentrations in the solution before and after the adsorption process, respectively. In addition, V is the volume of the solution (mL), and m (g) is the amount of sorbent.

## 4. Conclusions

Surface modifications of commercial ZrO_2_ nanoparticles by different mono- and di-carbamoyl phosphonic acid ligands have been developed and optimized through the direct grafting of Bronsted acid–base reaction, resulting in a series of inorganic–organic (hybrid) systems ZrO_2_-L**^n^**.

The performances of all prepared hybrid ZrO_2_-L**^n^** nano-absorbents were compared for the adsorption process of precious metals Au(III) and Pd(II) from highly acidic (HCl) solutions. Results obtained through this study showed that the higher the hydrophobic character of the mono-carbamoyl ligands, the better the extraction performance was, and Di-carbamoyl ligands have better adsorption efficiency than mono-carbamoyl ligands with higher selectivity towards Au(III).

The modified di-*N*,*N*-butyl carbamoyl pentyl phosphonic acid sample (ZrO_2_-L**^6^**) demonstrated remarkable selectivity to recover Au(III) and quantitative gold removal from the complex Pd mixture, Ni, Fe, Cu in real WEEE water with highly acidic and oxidizing environments. The adsorption followed the Langmuir adsorption model and pseudo-second-order model of thermodynamic and kinetic adsorption characteristics, respectively.

In terms of reusability, it was found that the adsorption efficiency of ZrO_2_-L**^6^** towards Au(III) was limited due to surface contamination using thiourea as a stripping agent. These could be solved by using sodium hydroxide as a cleaning agent after each adsorption/stripping cycles. However, this causes (partial) ligand degradation under basic conditions. A study to find an appropriate stripping agent is in progress.

## Figures and Tables

**Table 1:** Structures of the selected carbamoyl-based organic ligands to modify the nano-ZrO_2_ surface.

Group A: Mono-Carbamoyl Ligands			
N, N-Diethyl carbamoyl methyl phosphonic acid (DECMPA)	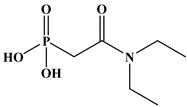	195 g.mol^−1^	L**^1^**
N, N-Dipropyl carbamoyl methyl phosphonic acid (DPCMPA)	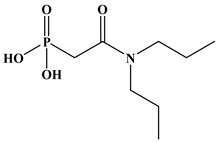	223 g.mol^−1^	L**^2^**
N,N-Dioctylcarbamoyl methyl phosphonic acid (DOCMPA)	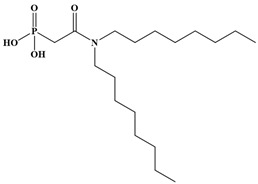	363 g.mol^−1^	L**^3^**
N,N-bis(2-ethylhexyl) carbamoyl methyl phosphonic acid (DEHCMPA)	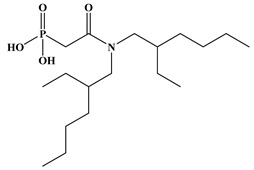	363 g.mol^−1^	L**^4^**
**Group B: Di-carbamoyl ligands**			
Di-N,N-butyl carbamoyl butyl phosphonic acid (DBCBPA)	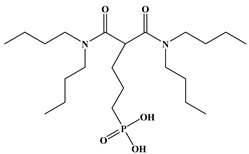	448 g.mol^−1^	L**^5^**
Di-N,N-butyl carbamoyl pentyl phosphonic acid (DBCPPA)	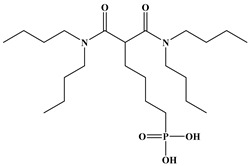	462 g.mol^−1^	L**^6^**

**Table 2:** Physico-chemical properties of ZrO_2_-L**^n^** nanomaterials.

Modified ZrO_2_-Ligand	Theoretical Weight Loss (%)	Experimental Weight Loss (%)	Grafted Density (L**^n^**/nm^2^)	Phosphorus Weight (%)	Surface Area (m^2^/g)
ZrO_2_	-	-	-	-	52.0 ± 4.0
ZrO_2_-L**^1^**	1.79	1.73	0.106	0.10	50.2 ± 0.4
ZrO_2_-L**^2^**	2.20	1.91	0.102	0.08	50.7 ± 1.4
ZrO_2_-L**^3^**	4.32	3.99	0.131	0.11	50.4 ± 0.7
ZrO_2_-L**^4^**	4.32	3.94	0.129	0.11	50.5 ± 1.0
ZrO_2_-L**^5^**	5.50	5.22	0.139	0.06	50.3 ± 0.6
ZrO_2_-L**^6^**	5.75	5.79	0.150	0.10	50.4 ± 0.7

**Table 3:** The distribution coefficient and separation factor values of Au(III) and Pd(II).

	Au(III)		Pd(II)	
	Distribution Coefficient (K_d_, mL g^−1^) × 10^3^	Separation Factor (SF_Au/pd_) Compared to Pd	Distribution Coefficient (K_d_, mL g^−1^) × 10^3^	Separation Factor (SF_Pd/Au_) Compared to Au
ZrO_2_	0.12	10.30	0.012	0.103
ZrO_2_-L**^1^**	0.13	3.71	0.035	0.270
ZrO_2_-L**^2^**	0.11	2.50	0.044	0.400
ZrO_2_-L**^3^**	0.90	16.40	0.055	0.061
ZrO_2_-L**^4^**	0.93	15.80	0.059	0.063
ZrO_2_-L**^5^**	1.27	18.14	0.070	0.055
ZrO_2_-L**^6^**	2.46	37.85	0.065	0.026

**Table 4:** Langmuir and freundlich isotherm parameters for the adsorption of Au(III) on ZrO_2_-L**^6^**.

Adsorbent	Adsorption of Au(III) Equilibrium Isotherm Models					
	Langmuir Isotherm			Freundlich Isotherm		
	q_max_ Capacity (mg/g)	Affinity Constant k_l_ (L/g)	R^2^ Correlation Coefficient	K_f_ Freundlich Constant (mg/g)	*n* Heterogeneity Coefficient (g/L)	R^2^ Correlation Coefficient
ZrO_2_-L**^6^**	6.54	0.1804	0.9892	1.65	2.89	0.9014

**Table 5:** Au(III) Adsorption kinetic models parameters using ZrO_2_-L**^6^** sample.

Kinetic Model	The Equilibrium Rate Constant (K)	qe (mg/g)	R^2^
pseudo-first order	0.0108	2.607	0.7565
pseudo-second order	0.0247	4.771	0.9985

**Table 6:** The weight percentage of Elemental analysis (% in weight).

Entry	Nano-Material	N%	C%	H%	S%
1	Commercial ZrO_2_	0.01	0.43	0.18	0.20
2	As-prepared ZrO_2_-L**^6^**	0.33	3.70	0.68	0.18
3	ZrO_2_-L**^6^**—5 cycles Adsorption/Stripping	0.58	3.82	0.43	0.51
4	Commercial ZrO_2_-NaOH	n.d	0.63	0.18	n.d
5	ZrO_2_-L**^6^**—5 cycles Adsorption/Stripping/NaOH (0.1 M)	0.04	5.83	0.21	n.d
6	ZrO_2_-L**^6^**—5 cycles Adsorption/Stripping/Na_2_CO_3_ (0.1 M)	0.04	2.50	0.39	n.d

* n.d. = non detected.

**Table 7:** Values of the initial concentration, the distribution coefficient, and the separation faction of Au compared to other competing metals present in real e-wastewater.

Metal	C_0_ (mg kg^−1^)	Kd (ml g^−1^) × 10^3^	SF(Au, M)
Au	10	1.820	-
Pd	6	0.004	455
Cu	7918	0.002	910
Ni	200	0.002	910
Fe	540	0.007	260

## Supplementary Materials

The following supporting information can be downloaded at: https://www.mdpi.com/article/10.3390/molecules28052219/s1, Figure S1: XRD spectra of commercial ZrO_2_ (10%wt. 100 nm) and modified ZrO_2_-L**^n^** nanoparticle; Figure S2: TEM images for (**A**) commertial ZrO_2_ (10%wt) and (**B**) modified ZrO_2_-L**^n^** nanoparticles; Figure S3: (**a**) Langmuir and (**b**) Freundlich isotherms for Au(III) adsorption on ZrO_2_-L**^6^**; Figure S4**:** (**a**) Pseudo second-order, and (**b**) pseudo first-order models for Au(III) adsorption kinetics; Table S1**:** ^31^P MAS chemical shift of phosphonic ligands (L**^n^**) and modified zirconia nanoparticles (ZrO_2_-L**^n^**).
