# Supplementary material for: New Carbamoyl Surface-Modified ZrO2 Nanohybrids for Selective Au Extraction from E-Waste
Source: Molecules. 2023 Feb 27;28(5):2219. doi: 10.3390/molecules28052219 (PMC10004478; doi:10.3390/molecules28052219)
Supplement: Supplementary file 1 [file molecules-28-02219-s001.zip › molecules-2237627-supplementary.pdf]

Supplementary Materials

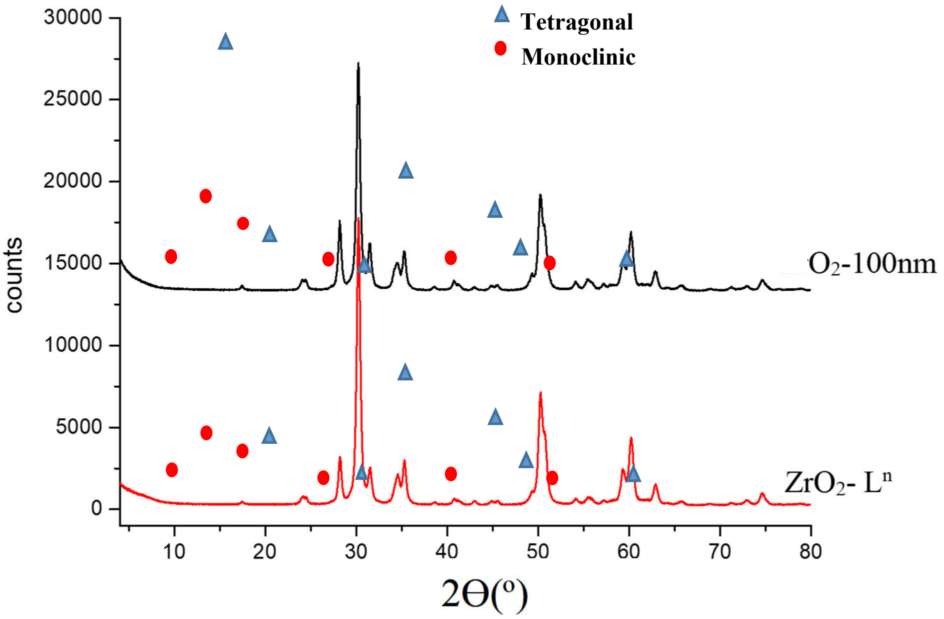

Figure S1. – XRD spectra of commercial ZrO_2 (10 %wt) and modified ZrO_2-L^n nanoparticle.

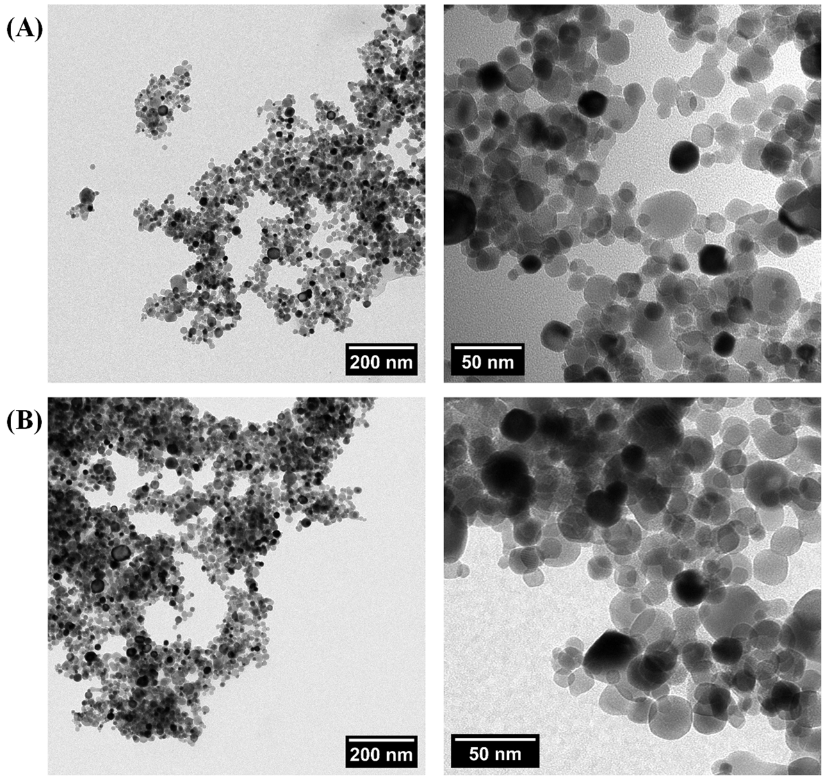

Figure S2. – TEM images for (A) commercial ZrO_2 (10 %wt) and (B) modified ZrO_2-L^n nanoparticles.

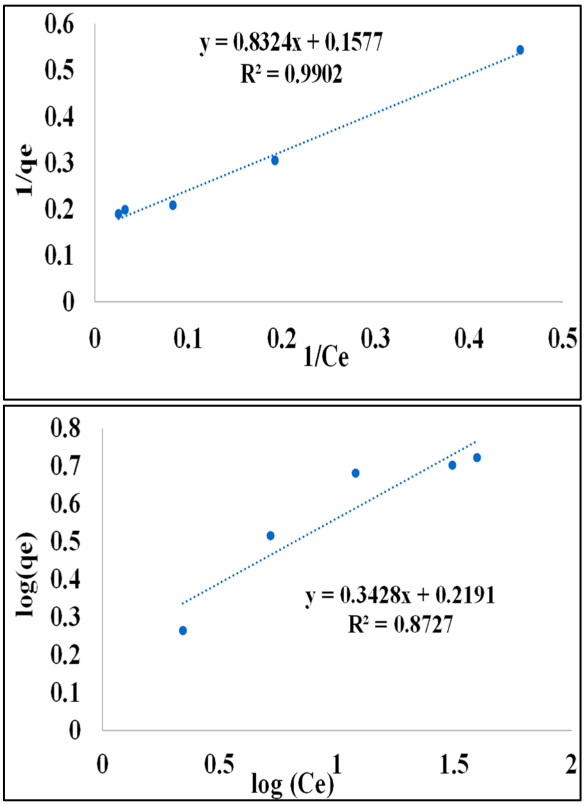

Figure S3. – a) Langmuir and b) Freundlich isotherms for Au(III) adsorption on ZrO_2-L[6].

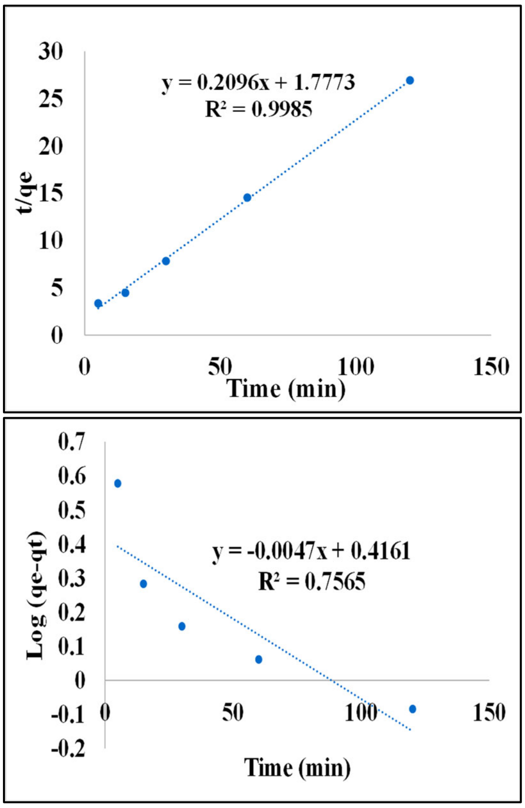

Figure S4. – a) Pseudo second order, and b) pseudo first-order models for Au(III) adsorption kinetics.

Table S1. ^{31}P MAS chemical shift of phosphonic ligands (L^n) and modified zirconia nanoparticles (ZrO_2-L^n).

Ligands	Chemical shift (ppm)	ZrO_2-L^n	Chemical shift (ppm)
L^1 = DECMPA	15.9	ZrO_2-L^1	11.6
L^2 = DPCMPA	15.2	ZrO_2- L^2	11.9
L^3 = DOCMPA	18.3	ZrO_2- L^3	11.0
L^4 = DEHCMPA	19.2	ZrO_2- L^4	11.8
L^5 = DBCBPA	29.2	ZrO_2- L^5	29.3 ; 22.2
L^6 = DBCPPA	29.7	ZrO_2- L^6	29.3 ; 22.8
